# Thirteenth Annual Meeting of the Medical Association of Central New York

**Published:** 1880-06

**Authors:** Charles E. Rider

**Affiliations:** Secretary


### Society Reports.

### THIRTEENTH ANNUAL MEETING OF THE MEDICAL ASSOCIATION OF CENTRAL NEW YORK,

HELD IN ROCHESTER, N. Y., MAY 18, 1880.

Dr. Frederick Hyde, of Cortland Co., President, in the chair. About one hundred delegates and permanent members were in attendance.

The meeting was called to order soon after 10 o'clock, A. M.

The minutes of the last meeting were read and adopted.

The President announced the standing committees, viz:

*On Credentials:*—Drs. Alfred Mercer, of Onondaga; J. N. Arnold, of Wayne; E. H. Howard, of Monroe.

*On Business* :—Drs. J. V. Kendall, of Onondaga; H. F. Bennett, of Ontario; James Chapman, of Orleans.

*On Reception* :—Drs. Darwin Colvin, of Wayne; E. W. Armstrong, of Orleans; C. S. Starr, of Monroe.

*On Ethics* :—Drs. E. M. Moore, of Monroe; W. W. Potter, of Genesee; G. W. Palmer, of Wyoming.

*On Publication* :—Dr. J. W. Palmer, of Ontario; H. C. Tompkins, of Orleans; The Secretry, *ex-officio*.

The Treasurer, Dr. Alfred Mercer, of Syracuse, made his annual report, which showed a balance of about $140.00 in the treasury.

He moved that a prize of——dollars be offered from the funds of the Association for the best original paper on some medical subject.

Dr. Rider moved as an amendment, that a committee of three be appointed by the chair, to consider the matter of prize essays, and to report during the afternoon session. Resolution as amended, carried.

The President appointed as such committee, Drs. Alfred Mercer, M. W. Townsend and Darwin Colvin.

Dr. E. W. Armstrong then read a report of four fatal cases of diphtheria in one family.

Dr. Frank Kenyon followed with a paper on the treatment of diphtheria, giving special prominence to salicylic acid.

Dr. J. W. Palmer considered an elevation of temperature as favorable, rather than an unfavorable symptom in diphtheria.

Dr. James Chapman read a paper on the treatment of fracture of both bones of the leg. He advised the bent position of the knee, open splints, and frequent inspection. Extension should be made in most cases. Rigid bandages should not be used too early or too long, since there is danger of obstruction of the veins.

Dr. Gregory Doyle then exhibited appliances of his own devising, for the treatment of club-foot and similar deformities. A patient was shown with the apparatus applied.

Dr. Hovey read a report of a remarkable ovarian cyst, presenting also the pathological specimen.

Dr. J. Dunn read a paper on some of the physiological effects and therapeutic uses of henbane, opium and belladonna, separate and combined.

Dr. E. M. Moore exhibited several subjects of resection of the joints and other similar operations.

Adjourned for dinner.

AFTERNOON SESSION.

After listening to the annual address of the President, the Association proceeded to the election of officers with the following result:

President, Dr. J. V. Kendall, of Onondaga; 1st Vice-President, Dr. C. M. Kingman, of Wayne; 2d Vice-President, Dr. L. J. Ames, of Livingston; Censor to Syracuse University, Dr. Darwin Colvin, of Wayne; Treasurer, Dr. Alfred Mercer, of Onondaga; Secretary, Dr. J. N. Arnold, of Wayne; Delegate to the British Medical Association, Dr. A. Clifford Mercer.

Dr. Dimon, of Auburn, was requested to write an obituary notice of the late Dr. Gilmore, of Fleming, and Dr. J. W. Palmer was appointed to do the same in the case of the late Dr. Webster, of East Bloomfield.

The following named were elected permanent members:

Monroe County, Drs. J. O. Roe, Wm. Eves. Oswego County, Drs. G. W. Nelson, Ella M. Whitaker. Onondaga County, Drs. H. B. Allen, I. H. Searls. Cayuga County, Drs. B. K. Hoxie, Frank Putman. Wyoming County, Drs. G. M. Palmer, J. A. Post.

The following named were made eligible for permanent membership:

Livingston County, Drs. J. W. Gray, F. H. Moyer. Genesee County, Drs. W. W. Potter, J. V. Mullen. Wayne County, Dr W. Putnam.

Dr. Mercer, Chairman of Committee on Prize Essays, reported as follows:

*Resolved;* That the Association offer a prize of twenty-five dollars, for the best paper on "The needed legal reforms, regulating the study and practice of medicine." Also a prize of twenty-five dollars for the best paper on any medical subject. Subject to be chosen by the writer. For either prize the writer must be a member of one of the societies represented in this Association.

A committee of three shall be appointed by the President, to report, at the next annual meeting, on the papers presented for the prizes.

The report and resolution were adopted.

A paper was then read by Dr. J. O. Roe, on nasal stenosis and its treatment by the galvano-cautery.

At 5 o'clock, P. M., the Association adjourned to meet in Rochester, November 16, 1880.

CHARLES E. RIDER, Secretary.

